# Positive Additive and Multiplicative Interactions among Clustered Components of Metabolic Syndrome with Type 2 Diabetes Mellitus among Brazilian Adolescent Students

**DOI:** 10.3390/nu14214640

**Published:** 2022-11-03

**Authors:** Rodolfo Deusdará, Amanda de Moura Souza, Moyses Szklo

**Affiliations:** 1 Faculty of Medicine, Campus Universitário Darcy Ribeiro, University of Brasilia, UnB, Asa Norte, Brasília 70910-900, DF, Brazil; 2 Institute for Studies in Public Health, Federal University of Rio de Janeiro, Avenida Horacio Macedo, S/N, Ilha do Fundão-Cidade Universitária, Rio de Janeiro 21941-598, RJ, Brazil; 3 Epidemiology Department, Johns Hopkins Bloomberg School of Public Health, 615 N. Wolfe Street, Baltimore, MD 21205, USA

**Keywords:** metabolic syndrome, diabetes mellitus, biomarkers, adolescent, synergism

## Abstract

Background: It is still controversial whether the joint effect of Metabolic syndrome (MetS) components is greater than that expected based on their independent effects, regarding type 2 diabetes mellitus in adolescents. We evaluated additive and multiplicative interactions between pair-wise combinations of metabolic syndrome components regarding type 2 diabetes mellitus. Methods: We studied 37,815 Brazilian adolescents from a national school-based survey, The Study of Cardiovascular Risk Factors in Adolescents (Portuguese acronym, ERICA). A Poisson regression model was used to calculate sex-, age-, obesity-, smoking status-, sedentary behavior-, physical inactivity-, alcoholic consumption- and socioeconomic status-adjusted prevalence ratios to evaluate both additive and multiplicative interactions. Results: In the comparison of observed and expected joint effects, relative excess risk due to additive interaction (RERI) for high triglycerides and low high-density lipoprotein-cholesterol, high triglycerides and elevated waist circumference, elevated waist circumference and low high-density lipoprotein-cholesterol and elevated waist circumference and high blood pressure were 2.53 (−0.41, 5.46), 2.86 (−2.89, 8.61), 1.71 (−1.05, 4.46) and 0.97 (0.15, 1.79), respectively, thus suggesting additive interactions. Multiplicative interactions for those pairs of components were also observed, as expressed by interaction ratios > 1.0. Conclusions: The joint presence of some of the components of MetS showed a greater association with the prevalence of type 2 diabetes mellitus in adolescents than expected from the sum of their isolated effects. From a public health perspective, preventing one of the components of the pairs that interact may result in a greater reduction in the prevalence of T2DM than focusing on an individual component that does not interact with another component.

## 1. Introduction

Type 2 diabetes mellitus (T2DM) is an important public health concern in adolescents, with a rising incidence occurring in parallel with increasing rates of childhood obesity [1] and its complications [2], such as renal disease and neuropathy [3]. Defects in insulin action and responses are present in the early stages of diabetes [2]. 

Childhood-onset T2DM is characterized by rapid progression to beta-cell failure [2]. The clinical presentation of diabetes has a broad spectrum, from mild symptoms to diabetic ketoacidosis [4]. Children are often diagnosed in mid to late puberty and most are obese. In T2DM’s mildest form, approximately one-third of children have shown to be asymptomatic (considering the typical diabetes symptoms) and their disease was detected by screening [5]. In addition, they usually have associated metabolic syndrome components, such as hypertension and dyslipidemia [5]. 

The term Metabolic Syndrome (MetS) is used to describe a complex pathophysiological connection between risk factors for T2DM and cardiovascular disease (CVD) [6,7]. According to the International Diabetes Federation (IDF), components of MetS are central obesity, triglycerides (TG), high-density lipoprotein-cholesterol (HDL-c), blood pressure (BP), and fasting plasma glucose (FPG) [8]. Those conditions share common pathways, mechanisms, and mediators [9]. Insulin resistance and central obesity are especially related to the development of MetS [8]. Due to rising trends of obesity and overweight in adolescents [10], the prevalence of MetS in adolescents is high in the U.S., the Middle East, and South America, particularly in Brazil and Colombia [7]. 

Several different criteria have been used to define MetS in adolescents [11], which may contribute to the variability of MetS prevalence: ranging from 0.3% in Colombia [12] to 26.4% in Iran [7,13]. Despite the apparent agreement on defining MetS components, there are differences in their cut-off points to define the syndrome.

Based on a literature review conducted in 2007, there were 46 different definitions of MetS in children and adolescents. Most of these definitions were adapted from the adult definition developed by the National Cholesterol Education Program [14]. In a recent systematic review, the most frequently used definition in children and adolescents was that from the IDF [8], followed by those of Cook [15], Ford [16], and Ferranti [17]. 

An important point pertaining to the definition of MetS as a syndrome is whether the co-occurrence of its components results in a greater risk of the outcome than would be expected by the sum of their independent effects. If the co-occurrence of MetS components does not imply a greater risk of diabetes than the mere sum of its independent effects, then we may question the term “syndrome” from the viewpoint of this construct [18]. Few studies have evaluated interactions among components of the metabolic syndrome [19,20,21,22,23,24]. In particular, little is known about interactions between pediatric MetS components and diabetes mellitus, which is the focus of our study. The presence of additive interaction is especially relevant to the prevention and public health in general, as the disease burden in the community should be measured in absolute, not relative, excess risk [25]. In other words, the presence of a positive additive interaction should be the framework of preventive strategies and public health policies, even when there is negative or null multiplicative interaction [26]. On the other hand, multiplicative interaction is preferable to assess causal relationships [27]. In the present study, the aim was to evaluate additive as well as multiplicative interactions among combinations of metabolic syndrome components, using T2DM as the outcome.

## 2. Materials and Methods

### 2.1. Study Design and Sample

The Study of Cardiovascular Risk Factors in Adolescents (Portuguese acronym, ERICA) was a national, multicenter, school-based, cross-sectional survey that included 75,000 adolescents aged 12 to 17 years from 1247 schools located in 122 municipalities with ≥100,000 inhabitants, conducted in 2013–2014. This analytic sample reflected the exclusion of 4 schools located in 2 municipalities, which refused participation [28]. Among the 72,508 students on the morning shift, of the total 102,327 eligible students, 37,815 (52,2%) adolescents from 111 municipalities had complete information from questionnaire data, anthropometrics, blood pressure, and fasting blood analyses [28]. 

The multistage sampling used stratification from 32 geographical areas (26 State capitals, Federal District, and 5 macro-regions). All local Ethics Committees approved the study procedures. A detailed description of the study design has been published [29,30].

### 2.2. Anthropometric and Blood Pressure Measurements

Waist circumference (WC) was measured with an anthropometric fiberglass tape, Sanny^®^. The measurement was taken at the medium point between the lower costal margin and the highest point of the iliac crest [30]. High WC was defined as values ≥ 90th percentile for those aged 10 to <16 years old; ≥90 cm for males, and ≥80 cm for females for those aged 16 years old and over [31].

Systolic and diastolic blood pressures were measured using the automatic oscillometric device Omron^®^ 705-IT (Omron Healthcare, Bannockburn, IL, USA). The appropriate cuff size was used, with the subject sitting with their feet flat on the ground [30]. Three consecutive measures were taken with intervals of 3 min. The average of the 2nd and 3rd BP readings was used to reduce the impact of reactivity on the BP values. High BP was defined as systolic or diastolic pressures of ≥130 or ≥85 mmHg, respectively [8].

### 2.3. Biochemical Assays

Only students in the morning classes (our analytic sample) had their blood samples collected [30]. All participants were instructed to fast overnight for 12 h before blood was collected the next morning. A thorough description of blood collection procedures as well as quantitative internal and external quality control procedures are available elsewhere [32]. Serum triglyceride, glucose, and HDL-c levels were measured using the enzymatic kinetics assay, hexoquinase method, and enzymatic colorimetric assay, respectively.

### 2.4. Metabolic Syndrome Definition 

The International Diabetes Federation (IDF) MetS’s definition considered elevated WC and the presence of two or more risk factors: elevated TG ≥ 150 mg/dL; and/or high glucose ≥ 100 mg/dL; and/or low HDL-c < 40 mg/dL (for boys aged 12–17 years and girls aged 12–15 years or HDL-c < 50 mg/dL in girls aged 16 and 17 years); and/or high BP ≥ 130/85 mmHg [31].

### 2.5. Outcome Definition

T2DM was defined by diagnosed and undiagnosed diabetes. Diagnosed diabetes was identified by whether a physician had told the participants that they had diabetes and whether participants used any diabetes treatment recommendation. Undiagnosed diabetes was defined as glucose ≥ 126 md/dL or HbA1C ≥ 6.5% and characterized by the absence of a physician’s identification [33]. Participants who declared they were using insulin and who were classified as having diabetes type 1 were not considered, as the focus of the study was T2DM. 

### 2.6. Statistical Analysis

Means and standard deviations for continuous variables with normal distributions were calculated. For other variables, medians and interquartile ranges were calculated. Normality was evaluated by the Shapiro-Wilk test.

ERICA’s complex sampling design and sampling weights were considered in the analyses [29].

Based on the definition of MetS by IDF, there were six pair-wise combinations for the evaluation of first-order interactions, as follows: HDL-c * TG; TG * WC; HDL-c * WC; BP * WC; BP * TG and BP * HDL-c for the association with T2DM. Since impaired fasting glucose is a strong predictor of T2DM and is included in the definition of metabolic syndrome, we decided not to use the glucose component to evaluate possible interactions between metabolic syndrome components and T2DM [18]. Our strategy to evaluate interactions was a Comparison of Observed and Expected Joint Effects [26].

The interaction was assessed on additive and multiplicative scales. While positive additive interaction is important to assess the public health need of an intervention, multiplicative interaction is preferable to assess causal relationships [27]. 

We used Poisson regression models to evaluate both additive and multiplicative interactions among pair-wise clusters of metabolic syndrome components (regarding T2DM) in the stratum formed by at least a third component, thus defining the presence of MetS. For example, when assessing the pair-wise combination of elevated WC and high TG, we examined their 1st order interaction in the stratum formed by high BP or low HDL-c, to meet MetS criteria. 

Additive interaction was measured by the Relative Excess Risk due to Interaction (RERI) [34]. RERI was obtained by the difference between the observed joint effect and the expected joint effect from the sum of each independent effect [34]. When RERI ≥ 0, a positive additive interaction is present. Multiplicative interaction was measured by the interaction ratio, obtained by dividing the observed joint effect by the multiplication of the independent (isolated) effects [27]. When the interaction ratio (IR) ≥ 1, a positive multiplicative interaction is present [26]. 

We used the delta method to calculate RERI and its 95% confidence interval [27], the interpretations of which were based on VanderWeele and Knol’s recommendations [27]. 

Covariates included in the multivariable-adjusted regression model included age, sex, obesity (which was based on age- and sex-specific BMI levels [35] with a Z-score ≥ +2), physical inactivity (<420 min per week), smoking status (≥1 cigarette smoked at least one day in the last 30 days), alcohol consumption (≥1 alcoholic drink at least one day in the last 30 days), sedentary behavior (≥3 h a day spent with television, video games or computer in an ordinary weekday), and socioeconomic status (defined by whether the adolescent attended public or private schools). 

All the analysis was conducted using STATA version 14 (StataCorp LP, College Station, TX, USA).

## 3. Results

### 3.1. Description of the Study Population

As seen in Table 1, the median age was 15 years for both boys and girls, and most adolescents were from public schools. The prevalence of unhealthy behavior varied widely from less than 5% for tobacco use to 63% for physical inactivity. In addition, almost 22% of adolescents consumed at least 1 alcoholic drink in the last 30 days, and approximately 40% of adolescents had sedentary behavior. About 3% of adolescents met the IDF criteria for MetS. Of those meeting the criteria, less than 5% had four or five components, with most adolescents having three components (Table 1). The prevalence of components of metabolic syndrome in descending order was low HDL-c, elevated WC, high BP, high TG, and high glucose (Table 1). Approximately 9% were obese based on age- and sex-specific BMI levels.

Approximately 3.3% had T2DM, with the majority having been identified by a physician (Table 1).

### 3.2. Comparing Observed and Expected Joint Effects

As seen in Table 2, the joint effects of two metabolic syndrome components were analyzed in each stratum formed by at least a third component. As observed in Table 2, in the multiplicative scale, the observed joint effect of high TG and low HDL was 3.08 times greater than the expected joint effect obtained by the formula shown in the Methods, section in the strata formed by elevated WC and/or High BP to meet MetS IDF criteria. In the additive scale, the RERI of 2.53 means that the prevalence ratio for T2DM in adolescents is 2.53 higher than in absence of interaction between High TG and Low HDL in the strata formed by elevated WC and/or High BP. The same pattern was seen in different two pairs of variables, namely, TG/WC and WC/HDL-c. The point estimates suggested interactions in both scales. However, the 95% confidence intervals for these interaction indices overlapped the null hypothesis (Table 2). In only one pair of components—WC and BP—the 95% CIs of both RERI and IR did not include the null value, RERI = 0.97 (0.15, 1.79) and IR = 3.71 (1.42, 9.70). High BP and high TG or low HDL-c showed results towards the null value, with RERI and interaction ratios of approximately 0 and 1, respectively.

**Figure 1:**
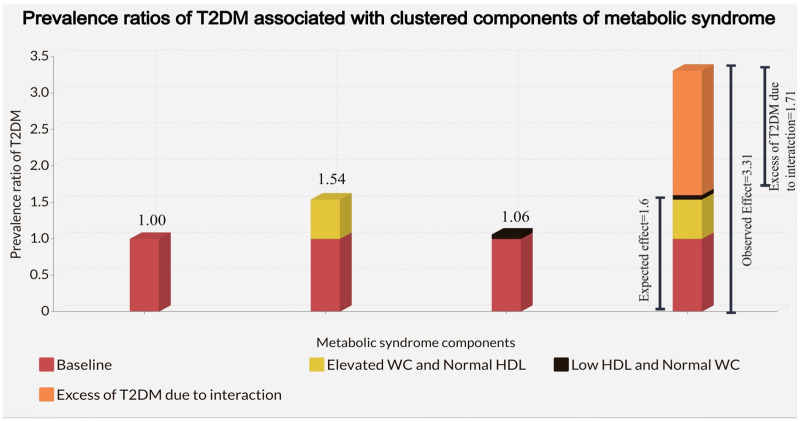
Adjusted Prevalence ratios of T2DM associated with the independent effect of elevated WC and low HDL and clustering of elevated WC and Low HDL in the stratum formed by high TG or high BP in 37,815 adolescents in the Study of Cardiovascular Risk Factors (ERICA, 2013–2014). Adjustment was made for age, sex, obesity, physical inactivity, sedentary behavior, alcohol consumption, smoking status, and socioeconomic status.

A schematical representation of RERI for the joint effect of elevated WC and low HDL in the stratum formed by high TG or high BP to meet the IDF criteria of MetS showing the difference between the expected joint effect obtained by the formula shown in Methods section and the observed joint effect in our study: (Figure 1) The excess of T2DM (orange) due to interaction for elevated WC and low HDL in the additive scale was the difference between the observed joint effect and the expected joint effect from the sum of each independent effect, 1.71.

## 4. Discussion

In our study, T2DM had a high prevalence of approximately 3.3%. The worldwide prevalence of T2DM in adolescents varied widely from approximately 0 to 5.1% [36,37,38,39]. According to the IDF Diabetes Atlas, the prevalence of Brazilian youth T2DM was as high as that in indigenous American and Mexican populations [40]. The ERICA study was the first school-based report in Latin America and indicates that T2DM had reached epidemic proportions in Brazilian adolescents [33]. Brazil is the fourth country in the number of incidents of T2DM in children and adolescents [41] and is the sixth country with adults living with diabetes in 2021 [42].

Our findings suggest that TG with WC or HDL-c, and WC with HDL-c interacted positively in both scales, with 95% CIs not overlapping the null value for the joint presence of both WC and BP. The fact that the 95% CIs of some additive and multiplicative indices (RERI and IR) overlap the null hypotheses (RERI = 0 and IR = 1.0) could be wrongly interpreted: that those interactions were absent. However, as the maximum likelihood is the indices’ point estimates, we prefer to say that those interactions are suggested rather than that there are no interactions [43,44].

Thus, in Brazilian adolescent students, who meet the MetS IDF criteria, prevention of high TG with elevated WC or Low HDL-c and elevated WC with low HDL-c or high BP might result in a greater reduction in the prevalence of T2DM than in those who only have one of the syndrome’s components.

We found that some independent associations of MetS components with T2DM had a PR below 1.0, including low HDL-c, high BP, and elevated WC. One possible explanation for those unexpected findings is that the cumulative effects of each risk factor in relation to T2DM may become evident only after several years of exposure in adolescents. Thus, long latent periods of those risk factors, the independent associations of which are difficult to identify in a cross-sectional study, may explain our results [45]. Another possible explanation is that self-report was the main source for the identification of T2DM in our study (93%), and it was not known when the diagnosis was made. Thus, adolescents with T2DM may have implemented healthy lifestyle measures, which may have resulted in attenuated prevalence ratios between risk factors and T2DM, a phenomenon known as “reverse causality” [46,47]. Another problem inherent to definitions of MetS is that its components are dichotomized, thus possibly resulting in a loss of information, which may have decreased the likelihood of finding precise associations [48].

The clustering of metabolic syndrome components is biologically plausible. WC is a strong marker of adiposity [49], which, in addition to insulin resistance, is a key determinant of MetS [8]. Visceral obesity contributes to insulin resistance and is also related to a dyslipidemic profile [50]. The increased flux of free fatty acids (FFAs) from adipose tissue to the liver promotes an increased triglyceride synthesis in the liver. The accumulation of intracellular lipid metabolites in the liver appears to cause hepatic insulin resistance [50]. In insulin-resistance settings, the development of hypertension is due to the loss of the vasodilator effect of insulin and the vasoconstriction caused by FFAs [51]. Additional mechanisms include increased sympathetic activation and sodium reabsorption in the kidneys [51].

Our study has demonstrated the key role that WC plays in interacting with other metabolic syndrome components with regard to prevalent T2DM. The findings, combined with those of previous studies, suggest that, pending confirmation in longitudinal studies, screening for diabetes should include WC measurement [52,53,54,55]. In addition, based on the literature, high consumption of both ultra-processed foods and sugar-sweetened beverages has been associated with metabolic syndrome in adolescents [56,57]. First-line approach to treat abdominal obesity is based on lifestyle interventions, -namely, moderate/vigorous physical activity and reduction of sugar-sweetened beverages and high-fat, high-sodium, and processed foods; and an increase in intake of fruits, vegetables, and fiber along with portion control education [58,59,60].

It is difficult to compare our results with those of other studies because they used different MetS criteria, [61,62,63] and included BMI rather than waist circumference in the definition of the syndrome [61,62]. In a pooled study using data from two prospective studies (The Bogalusa Heart Study and the Cardiovascular Risk Study), children and adolescents who met the pediatric metabolic syndrome criteria were more likely to develop T2DM over a mean 24-year period than those free of MetS. High BMI and hypertriglyceridemia were strongly predictive of an increased risk of T2DM in this pooled cohort [62]. In another prospective study, US children and adolescents with metabolic syndrome were found to be more likely than their peers to develop T2DM 25–30 years later [61]. In the Tehran Lipid and Glucose Study of 11-to 19-year-old adolescents, who met Cook’s metabolic syndrome definition [15], combinations of high WC and high TG or high BP, and high TG and BP were associated with early adulthood T2DM [60]. However, the authors of this study did not examine or test interactions, nor did they fully adjust for important confounders, such as puberty and physical activity. All these studies evaluated the predictive, but not the construct validity of MetS [61,62,63].

Few studies have evaluated interactions of metabolic syndrome components with regard to cardiovascular disease outcomes. In the Atherosclerosis Risk in Communities (ARIC) study, the observed joint association of hypertriglyceridemia and hypertension with an excess carotid intimal-medial thickness (IMT) was much stronger than that expected [24]. Other studies, however, have failed to show interactions between MetS components when evaluating IMT as the outcome in both adolescents [64] and adults [21,22], in relation to acute myocardial infarction in adults [65].

Our study has several strengths, including rigorous quality assurance and control. Additionally, to our knowledge, this is the largest study of MetS in adolescents based on a country-wide representative sample. Furthermore, ERICA’s large sample size allowed stratification of the MetS components for evaluation of interactions.

However, our study also has some limitations. This is a cross-sectional study and, thus, subjected to both selection and temporal biases, although the representativeness of the study population and the young ages of the participants make those biases less likely. In addition, as in all observational studies, residual confounding may have occurred. Another potential limitation is that only two categories of MetS components were considered, perhaps leading to a loss of information, though dichotomization is useful in clinical practice as it allows distinguishing “abnormal” from “normal” results [21]. It is difficult to compare data from the IDF definition [8] with those of other modified NCEP criteria (Cook [15], Ford [16], and Ferranti [17]) for two main reasons: (1) The IDF [8] requires the presence of abdominal obesity for the metabolic syndrome definition, whereas in the other modified NCEP definitions [15,16,17] abdominal obesity is not a mandatory criterion; (2) Regarding the other MetS components: the IDF MetS definition [8] for hypertension is SBP ≥ 130 or DBP ≥ 85 mmHg and dyslipidemia (e.g., triglycerides ≥ 150 mg/dL and HDL < 40 mg/dL for boys or HDL < 50 mg/dL for girls) was based on cutoff values for adults, whereas in the other modified NCEP criteria [15,16,17], hypertension was defined as age- and sex-specific BP ≥ 90th percentile and lower cutoff values for dyslipidemia (e.g., triglycerides ≥ 110 mg/dL [15,16]). In addition, it has been reported that the use of the IDF MetS definition is more appropriate because it is more specific and may avoid false positives [66]. Another limitation of the study was that T2DM was identified mostly by self-report, and we did not measure post-prandial glucose in the definition of T2DM, likely resulting in some degree of misclassification. Lastly, the 52% response rate of adolescents who had complete information and from whom we collected blood samples may impact the internal validity and, thus, limit the external validity of our results. Non-participants were more likely to be male (52.6% vs. 44.6% participants) and aged between 15 and 17 years (64.3% vs. 54.1% participants). However, selection bias is not likely, as the adolescents were not informed of the hypothesis about interactions of metabolic syndrome components.

## 5. Conclusions

The joint presence of high TG with elevated WC or Low HDL-c and elevated WC with low HDL-c or high BP seemed to result in both additive and multiplicative interactions regarding T2DM in our study, which is consistent with the construct validity of the “syndrome” definition. From a public health perspective, preventing one of the components of the pairs that interact may result in a greater reduction in the prevalence of T2DM than focusing on an individual component that does not interact with another component. Future studies should use a prospective approach and evaluate the presence of interactions in clusters of continuous components of the MetS regarding T2DM.

## Figures and Tables

**Table 1:** Characteristics of 37,815 study participants in the Study of Cardiovascular Risk Factors in Adolescents (ERICA, 2013–2014).

Variables	n			
Continuous		Median	1ºQ 3ºQ	
Age	37,815	15	13	16
Categorical		(%)	95% Confidence intervals	
Female	22,682	50.2		
Smoking (≥1 cigarette smoked in the last 30 days)	1406	4.2	3.8	4.7
Alcohol consumption (≥1 drink in the last 30 days)	7685	21.6	20.3	23.0
Sedentary behavior ^γ^	14,133	40.5	38.9	42.1
Physical inactivity (≤ 420 min per week)	24,713	62.7	61.7	63.8
Obesity (%)	3097	9.2	8.5	10.0
Public Schools	27,990	77.8	72.4	82.3
Metabolic syndrome *	861	2.6	2.3	2.9
Components of metabolic syndrome				
One component	13,025	33.7	31.9	35.6
Two components	3390	9.5	8.5	10.6
Three components	825	2.5	2.1	2.9
Four components	134	0.4	0.3	0.6
Five components	9	0.1	0.0	0.2
Metabolic syndrome components				
Elevated waist circumference ***	4386	12.6	11.6	13.7
High blood pressure ^#^	2677	8.2	7.6	8.9
High glucose ^δ^	1147	4.1	3.5	4.8
High triglycerides ^λ^	1712	4.6	4.1	5.1
Low HDL-c ** (%)	13,076	32.7	30.3	35.2
Type 2 Diabetes Mellitus ^Γ^	1227	3.28	2.91	3.69
Identified by a physician ^θ^	1126	3.05	2.69	3.46
Undiagnosed diabetes ^φ^	101	0.23	0.16	0.31

* Definition of metabolic syndrome: elevated waist circumference (values ≥ 90th percentile for those aged 10 to 16 years old; ≥90 cm for males and ≥80 cm for females for those aged 16 years and over) and the presence of two or more risk factors (high triglycerides ≥ 150 mg/dL, and/or high glucose ≥ 100 mg/dL, and/or low HDL-c < 40 mg/dL or hdl < 50 mg/dL in girls aged 16 and 17 and/or high blood pressure ≥ 130/85 mmHg). *** values ≥ 90th percentile for those aged 10 to 16 years old; ≥90 cm for males and ≥80 cm for females for those aged 16 years or older. ** <40 mg/dL or hdl < 50 mg/dL in girls aged 16 and 17. ^γ^ ≥ 3 h a day spent with television, video games, or computer in an ordinary weekday. ^#^ ≥130/85 mmHg. ^λ^ ≥150 mg/dL. ^δ^ ≥100 mg/dL. ^Γ^ defined by self-report diabetes, glucose ≥ 126 md/dL or HbA1c ≥ 6.5%. ^θ^ identified by whether a physician had told the participants that they had diabetes. ^φ^ fasting glucose ≥ 126 md/dL or HbA1c ≥ 6.5% and absence of a physician’s identification for diabetes.

**Table 2:** Adjusted ^‡^ Prevalence Ratios (PR) to assess the interaction between pair-wise clusters of metabolic syndrome components with type 2 diabetes mellitus ^Γ^ as the outcome, in the stratum formed by at least a third metabolic syndrome variable ^δ^ in 37,815 adolescents in the Study of Cardiovascular Risk Factors (ERICA, 2013–2014).

			Interaction Measures
Elevated WC * and/or High BP ^#^			
	Normal HDL-c	Low HDL-c **	
Normal TG	1	PR = 0.83 (0.52, 1.31)	RERI _TG * HDL-c_ = 2.53 (−0.41, 5.46)
High TG ^λ^	PR = 1.53 (0.58, 4.04)	PR = 3.88 (1.88, 8.01)	Interaction ratio _TG*HDL-c_ = 3.08 (0.90, 10.55)
Low HDL-c ** or High BP ^#^			
	Normal WC	Elevated WC *	
Normal TG	1	PR = 1.00 (0.53, 1.91)	RERI _TG * WC_ = 2.86 (−2.89, 8.61)
High TG ^λ^	PR = 1.89 (0.87, 4.14)	PR = 4.76 (1.42, 15.95)	Interaction ratio _TG*WC_ = 2.51 (0.75, 8.44)
High TG ^λ^ or High BP ^#^			
	Normal HDL-c	Low HDL-c **	
Normal WC	1	PR = 1.06 (0.45, 2.47)	RERI _WC * HDL-c_ = 1.71 (−1.05, 4.46)
Elevated WC *	PR = 1.54 (0.63, 3.78)	PR = 3.31 (1.18, 9.25)	Interaction ratio _WC*HDL-c_ = 2.03 (0.59, 7.01)
High TG ^λ^ or Low HDL-c **			
	Normal BP	High BP ^#^	
Normal WC	1	PR = 0.41 (0.22, 0.75)	RERI _WC * BP_ = 0.97 (0.15, 1.79)
Elevated WC *	PR = 0.73 (0.36, 1.47)	PR = 1.10 (0.46, 2.68)	Interaction ratio _WC * BP_ = 3.71 (1.42, 9.70)
Elevated WC * and/ or Low HDL-c **			
	Normal TG	High TG ^λ^	
Normal BP	1	PR = 3.29 (1.58, 6.83)	RERI _BP * TG_ = 0.50 (−4.17, 5.18)
High BP ^#^	PR = 1.02 (0.55, 1.89)	PR = 3.81 (1.26, 11.58)	Interaction ratio _BP * TG_ = 1.14 (0.27, 4.80)
Elevated WC * and/or High TG ^λ^			
	Normal HDL-c	Low HDL-c **	
Normal BP	1	PR = 1.02 (0.63, 1.64)	RERI _BP * HDL-c_ = 0.27 (−0.99, 1.53)
High BP ^#^	PR = 0.92 (0.41, 2.01)	PR = 1.21 (0.54, 2.71)	Interaction ratio _BP * HDL-c_ = 1.30 (0.39, 4.35)

^‡^ Adjusted for age, sex, obesity, physical inactivity, sedentary behavior, alcohol consumption, smoking status, and socioeconomic status. * values ≥ 90th percentile for those aged 10 to 16 years old; ≥90 cm for males and ≥80 cm for females for those aged 16 years and over. ** <40 mg/dL or hdl-c < 50 mg/dL in girls aged 16 and 17. ^#^ ≥130/85 mmHg. **^λ^** ≥150 mg/dL. ^Γ^ Identified by a physician, self-report treatment, glucose ≥ 126 md/dL or HbA1c ≥ 6.5%. ^δ^ To meet MetS criteria, we stratified by a third variable or WC and/or the fourth variable.

## Data Availability

The data presented in this study are available upon request from the corresponding author.

## References

[1] Liu L.L., Lawrence J., Davis C., Liese A.D., Pettitt D.J., Pihoker C., Dabelea D., Hamman R., Waitzfelder B., Kahn H. (2010). Prevalence of overweight and obesity in youth with diabetes in USA: The SEARCH for Diabetes in Youth study. Pediatr. Diabetes.

[2] Savic Hitt T.A., Katz L.E.L. (2020). Pediatric Type 2 Diabetes: Not a Mini Version of Adult Type 2 Diabetes. Endocrinol. Metab. Clin. N. Am..

[3] Dart A.B., Martens P.J., Rigatto C., Brownell M.D., Dean H.J., Sellers E.A. (2014). Earlier Onset of Complications in Youth With Type 2 Diabetes. Diabetes Care.

[4] Tfayli H., Arslanian S. (2009). Pathophysiology of type 2 diabetes mellitus in youth: The evolving chameleon. Arq. Bras. Endocrinol. Metabol..

[5] Reinehr T. (2005). Clinical presentation of type 2 diabetes mellitus in children and adolescents. Int. J. Obes..

[6] Alberti K.G.M.M., Eckel R.H., Grundy S.M., Zimmet P.Z., Cleeman J.I., Donato K.A., Fruchart J., James W.P.T., Loria C.M., Smith S.C. (2009). Harmonizing the metabolic syndrome: A joint interim statement of the international diabetes federation task force on epidemiology and prevention; National heart, lung, and blood institute; American heart association; World heart federation; International atherosclerosis society; And international association for the study of obesity. Circulation.

[7] Reisinger C., Nkeh-Chungag B.N., Fredriksen P.M., Goswami N. (2021). The prevalence of pediatric metabolic syndrome—A critical look on the discrepancies between definitions and its clinical importance. Int. J. Obes..

[8] Alberti K.G.M.M., Zimmet P., Shaw J. (2006). Metabolic syndrome—A new world-wide definition. A consensus statement from the International Diabetes Federation. Diabet. Med..

[9] Huang P.L. (2009). A comprehensive definition for metabolic syndrome. Company of Biologists; DMM Dis. Model. Mech..

[10] Bentham J., Di Cesare M., Bilano V., Bixby H., Zhou B., Stevens G.A., Ezzati M., Riley L.M., Taddei C., Hajifathalian K. (2017). Worldwide trends in body-mass index, underweight, overweight, and obesity from 1975 to 2016: A pooled analysis of 2416 population-based measurement studies in 128·9 million children, adolescents, and adults. Lancet.

[11] Miller J.M., Kaylor M.B., Johannsson M., Bay C., Churilla J.R. (2014). Prevalence of metabolic syndrome and individual criterion in US adolescents: 2001–2010 national health and nutrition examination survey. Mary Ann Liebert Inc. Metab. Syndr. Relat. Disord..

[12] Ramírez-Vélez R., Anzola A., Martinez-Torres J., Vivas A., Tordecilla-Sanders A., Prieto-Benavides D., Izquierdo M., Correa-Bautista J.E., Garcia-Hermoso A. (2016). Metabolic Syndrome and Associated Factors in a Population-Based Sample of Schoolchildren in Colombia: The FUPRECOL Study. Metab. Syndr. Relat. Disord..

[13] Asghari G., Eftekharzadeh A., Hosseinpanah F., Ghareh S., Mirmiran P., Azizi F. (2017). Instability of different adolescent metabolic syndrome definitions tracked into early adulthood metabolic syndrome: Tehran Lipid and Glucose Study (TLGS). Pediatr. Diabetes.

[14] Ford E.S., Li C. (2008). Defining the Metabolic Syndrome in Children and Adolescents: Will the Real Definition Please Stand Up?. J. Pediatr..

[15] Cook S., Weitzman M., Auinger P., Nguyen M., Dietz W.H. (2003). Prevalence of a metabolic syndrome phenotype in adolescents: Findings from the third National Health and Nutrition Examination Survey, 1988–1994. Arch. Pediatr. Adolesc. Med..

[16] Ford E.S., Ajani U.A., Mokdad A.H. (2005). The metabolic syndrome and concentrations of C-reactive protein among U.S. youth. Diabetes Care.

[17] de Ferranti S.D., Gauvreau K., Ludwig D.S., Neufeld E.J., Newburger J.W., Rifai N. (2004). Prevalence of the metabolic syndrome in American adolescents: Findings from the Third National Health and Nutrition Examination Survey. Circulation.

[18] Kahn R., Buse J., Ferrannini E., Stern M. (2005). The metabolic syndrome: Time for a critical appraisal—Joint statement from the American Diabetes Association and the European Association for the Study of Diabetes. American Diabetes Association; Diabetes Care..

[19] Bonora B.M., Marescotti M., Marcuzzo G., Avogaro A., Fadini G.P. (2015). Synergistic interactions among metabolic syndrome components and homeostasis model assessment of insulin resistance in a middle-aged general population over time. Metab. Syndr. Relat. Disord..

[20] Vaidya D., Szklo M., Liu K., Schreiner P.J., Bertoni A.G., Ouyang P. (2007). Defining the metabolic syndrome construct: Multi-Ethnic Study of Atherosclerosis (MESA) cross-sectional analysis. Diabetes Care.

[21] Fadini G.P., Coracina A., Inchiostro S., Tiengo A., Avogaro A., de Kreutzenberg S.V. (2008). A stepwise approach to assess the impact of clustering cardiometabolic risk factors on carotid intima-media thickness: The metabolic syndrome no-more-than-additive. Eur. J. Prev. Cardiol..

[22] Baldassarre D., Werba J.P., Castelnuovo S., Frigerio B., Amato M., Ravani A., Veglia F., Sirtori C.R., Tremoli E. (2010). The metabolic syndrome predicts carotid intima-media thickness no better than the sum of individual risk factors in a lipid clinic population. Atherosclerosis.

[23] Inchiostro S., Fadini G.P., de Kreutzenberg S.V., Citroni N., Avogaro A. (2007). Is the Metabolic Syndrome a Cardiovascular Risk Factor Beyond Its Specific Components?. J. Am. Coll. Cardiol..

[24] Golden S.H., Folsom A.R., Coresh J., Richey Sharrett A., Szklo M., Brancati F. (2002). Risk factor groupings related to insulin resistance and their synergistic effects on subclinical atherosclerosis: The Atherosclerosis Risk in Communities Study. Diabetes.

[25] Rothman K.J., Greenland S., Walker A.M. (1980). Concepts of interaction. Am. J. Epidemiol..

[26] Szklo M., Nieto F.J. (2019). Defining and Assessing Heterogeneity of Effects: Interaction. Epidemiology Beyond The Basics, 4th Ed.

[27] VanDerWeele T.J., Knol M.J. (2014). A tutorial on interaction. Epidemiol. Method.

[28] Da Silva T.L.N., Klein C.H., De Moura Souza A., Barufaldi L.A., De Azevedo Abreu G., Kuschnir M.C.C., de Vasconcellos M.T.L., Bloch K.V. (2016). Response rate in the study of cardiovascular risks in adolescents—ERICA. Rev. Saude Publica.

[29] de Vasconcellos M.T.L., do Silva P.L.N., Szklo M., Kuschnir M.C.C., Klein C.H., de Abreu G.A., Barufaldi L.A., Bloch K.V. (2015). Sampling design for the Study of Cardiovascular Risks in Adolescents (ERICA). Cad. Saude Publica.

[30] Bloch K.V., Szklo M., Kuschnir M.C.C., de Abreu G.A., Barufaldi L.A., Klein C.H., de Vasconcelos Maurício T.L., da Veiga Glória V., Figueiredo V.C., Dias A. (2015). The study of cardiovascular risk in adolescents—ERICA: Rationale, design and sample characteristics of a national survey examining cardiovascular risk factor profile in Brazilian adolescents. BMC Public Health.

[31] Alberti S.G., Zimmet P. (2007). The IDF Consensus definition of the Metablic Syndrome in Children and Adolescents. Int. Diabetes Fed..

[32] Cureau F.V., Bloch K.V., Henz A., Schaan C.W., Klein C.H., de Oliveira C.L., Giannini D.T., de Leon E.B., Abreu G.d., Telo G.H. (2017). Challenges for conducting blood collection and biochemical analysis in a large multicenter school-based study with adolescents: Lessons from ERICA in Brazil. Cad. Saude Publica.

[33] Telo G.H., Cureau F.V., Szklo M., Bloch K.V., Schaan B.D. (2019). Prevalence of type 2 diabetes among adolescents in Brazil: Findings from Study of Cardiovascular Risk in Adolescents (ERICA). Pediatr. Diabetes.

[34] Rothman K.J. (1986). Modern Epidemiology.

[35] Onis MDe Onyango A.W., Borghi E., Siyam A., Siekmann J. (2007). Development of a WHO growth reference for school-aged children and adolescents. Bull. World Health Organ..

[36] Lawrence J.M., Divers J., Isom S., Saydah S., Imperatore G., Pihoker C., Marcovina S.M., Mayer-Davis E.J., Hamman R.F., Dolan L. (2021). Trends in Prevalence of Type 1 and Type 2 Diabetes in Children and Adolescents in the US, 2001–2017. JAMA.

[37] Fagot-Campagna A., Pettitt D.J., Engelgau M.M., Ríos Burrows N., Geiss L.S., Valdez R., Beckles G.L., Saaddine J., Gregg E.W., Williamson D.F. (2000). Type 2 diabetes among North adolescents: An epidemiologic health perspective. J. Pediatr..

[38] Kahkoska A.R., Dabelea D. (2021). Diabetes in Youth: A Global Perspective. Endocrinol. Metab. Clin. N. Am..

[39] Fazeli Farsani S., Van Der Aa M.P., Van Der Vorst M.M.J., Knibbe C.A.J., De Boer A. (2013). Global trends in the incidence and prevalence of type 2 diabetes in children and adolescents: A systematic review and evaluation of methodological approaches. Diabetologia.

[40] International Diabetes Federation (2021). International Diabetes Federation. IDF Diabetes Atlas, 10th ed.

[41] Wu H., Patterson C.C., Zhang X., Ghani R.B.A., Magliano D.J., Boyko E.J., Ogle G.D., Luk A.O.Y. (2022). Worldwide estimates of incidence of type 2 diabetes in children and adolescents in 2021. Diabetes Res. Clin. Pract..

[42] Sun H., Saeedi P., Karuranga S., Pinkepank M., Ogurtsova K., Duncan B.B., Stein C., Basit A., Chan J.C.N., Mbanya J.C. (2022). IDF Diabetes Atlas: Global, regional and country-level diabetes prevalence estimates for 2021 and projections for 2045. Diabetes Res. Clin. Pract..

[43] Szklo M., Nieto F.J. (2019). Communicating Results of Epidemiologic Studies. Epidemiology Beyond The Basics.

[44] Rothman K.J. (2014). Six persistent research misconceptions. J. Gen. Intern. Med..

[45] Pollock B.D., Chen W., Harville E.W., Shu T., Fonseca V., Mauvais-Jarvis F., Kelly T.N., Bazzano L.A. (2018). Differential sex effects of systolic blood pressure and LDL-C on Type 2 diabetes: Life-course data from the Bogalusa Heart Study. J. Diabetes.

[46] Ginsberg H.N., Zhang Y.L., Hernandez-Ono A. (2005). Regulation of Plasma Triglycerides in Insulin Resistance and Diabetes. Arch. Med. Res..

[47] Lee S., Ryu K.S., Ye H., Kang J., You N.Y., Choi K.S., Hwangbo Y., Lee J.W., Cha H.S. (2021). Risk Factors of Undiagnosed Diabetes Mellitus among Korean Adults: A National Cross-Sectional Study Using the KNHANES Data. Public Health.

[48] Royston P., Altman D.G., Sauerbrei W. (2006). Dichotomizing continuous predictors in multiple regression: A bad idea. Stat. Med..

[49] Janssen I., Heymsfield S.B., Allison D.B., Kotler D.P., Ross R. (2002). Body mass index and waist circumference independently contribute to the prediction of nonabdominal, abdominal subcutaneous, and visceral fat. Am. J. Clin. Nutr..

[50] Rader D.J. (2007). Effect of Insulin Resistance, Dyslipidemia, and Intra-abdominal Adiposity on the Development of Cardiovascular Disease and Diabetes Mellitus. Am. J. Med..

[51] Tripathy D., Mohanty P., Dhindsa S., Syed T., Ghanim H., Aljada A., Dandona P. (2003). Elevation of free fatty acids induces inflammation and impairs vascular reactivity in healthy subjects. Diabetes.

[52] American Diabetes Association (2002). Screening for Diabetes. Diabetes Care.

[53] Wallace A.S., Wang D., Shin J.I., Selvin E. (2020). Screening and diagnosis of prediabetes and diabetes in us children and adolescents. Pediatrics.

[54] Savva S.C., Tornaritis M., Savva M.E., Kourides Y., Panagi A., Silikiotou N., Georgiou C., Kafatos A. (2000). Waist circumference and waist-to-height ratio are better predictors of cardiovascular disease risk factors in children than body mass index. Int. J. Obes. Relat. Metab. Disord. J. Int. Assoc. Study Obes..

[55] Ma L., Cai L., Deng L., Zhu Y., Ma J., Jing J., Chen Y. (2016). Waist Circumference is Better Than Other Anthropometric Indices for Predicting Cardiovascular Disease Risk Factors in Chinese Children—A Cross-Sectional Study in Guangzhou. J. Atheroscler. Thromb..

[56] Tavares L.F., Fonseca S.C., Garcia Rosa M.L., Yokoo E.M. (2012). Relationship between ultra-processed foods and metabolic syndrome in adolescents from a Brazilian Family Doctor Program. Public Health Nutr..

[57] Chan T.F., Lin W.T., Huang H.L., Lee C.Y., Wu P.W., Chiu Y.W., Huang C., Tsai S., Lin C., Lee C. (2014). Consumption of sugar-sweetened beverages is associated with components of the metabolic syndrome in adolescents. Nutrients.

[58] Fornari E., Maffeis C. (2019). Treatment of Metabolic Syndrome in Children. Front. Endocrinol..

[59] DeBoer M.D. (2019). Assessing and Managing the Metabolic Syndrome in Children and Adolescents. Nutrients.

[60] Styne D.M., Arslanian S.A., Connor E.L., Farooqi I.S., Murad M.H., Silverstein J.H., Yanovski J.A. (2017). Pediatric Obesity—Assessment, Treatment, and Prevention: An Endocrine Society Clinical Practice Guideline. J. Clin. Endocrinol. Metab..

[61] Morrison J.A., Friedman L.A., Wang P., Glueck C.J. (2008). Metabolic Syndrome in Childhood Predicts Adult Metabolic Syndrome and Type 2 Diabetes Mellitus 25 to 30 Years Later. J. Pediatr..

[62] Magnussen C.G., Koskinen J., Chen W., Thomson R., Schmidt M.D., Srinivasan S.R., Kivimäki M., Mattsson N., Kähönen M., Laitinen T. (2010). Pediatric metabolic syndrome predicts adulthood metabolic syndrome, subclinical atherosclerosis, and type 2 diabetes mellitus but is no better than body mass index alone: The Bogalusa Heart Study and the Cardiovascular Risk in Young Finns Study. Circulation.

[63] Asghari G., Hasheminia M., Heidari A., Mirmiran P., Guity K., Shahrzad M.K., Azizi F., Hadaegh F. (2021). Adolescent metabolic syndrome and its components associations with incidence of type 2 diabetes in early adulthood: Tehran lipid and glucose study. Diabetol. Metab. Syndr..

[64] Reinehr T., Wunsch R., Pütter C., Scherag A. (2013). Relationship between Carotid Intima-Media Thickness and Metabolic Syndrome in Adolescents. J. Pediatr..

[65] Mente A., Yusuf S., Islam S., McQueen M.J., Tanomsup S., Onen C.L., Rangarajan S., Gerstein H.C., Anand S.S., INTERHEART Investigators (2010). Metabolic syndrome and risk of acute myocardial infarction a case-control study of 26,903 subjects from 52 countries. J. Am. Coll. Cardiol..

[66] Kuschnir M.C.C., Bloch K.V., Szklo M., Klein C.H., Barufaldi L.A., Abreu G.A., Schaan B., da Veiga G.V., da Silva T.L.N., de Vasconcellos M.T.L. (2016). ERICA: Prevalence of metabolic syndrome in Brazilian adolescents. Rev. Saude Publica.

